# Measurement of fetal fraction in cell-free DNA from maternal plasma using a panel of insertion/deletion polymorphisms

**DOI:** 10.1371/journal.pone.0186771

**Published:** 2017-10-30

**Authors:** Angela N. Barrett, Li Xiong, Tuan Z. Tan, Henna V. Advani, Rui Hua, Cecille Laureano-Asibal, Richie Soong, Arijit Biswas, Niranjan Nagarajan, Mahesh Choolani

**Affiliations:** 1 Department of Obstetrics and Gynaecology, Yong Loo Lin School of Medicine, National University of Singapore, Singapore, Singapore; 2 Department of Gynecology & Obstetrics, Nanfang Hospital, Southern Medical University, Guangzhou, People's Republic of China; 3 Cancer Science Institute, National University of Singapore, Singapore, Singapore; 4 Genome Institute of Singapore, Singapore, Singapore; Chinese University of Hong Kong, HONG KONG

## Abstract

**Objective:**

Cell-free DNA from maternal plasma can be used for non-invasive prenatal testing for aneuploidies and single gene disorders, and also has applications as a biomarker for monitoring high-risk pregnancies, such as those at risk of pre-eclampsia. On average, the fractional cell-free fetal DNA concentration in plasma is approximately 15%, but can vary from less than 4% to greater than 30%. Although quantification of cell-free fetal DNA is straightforward in the case of a male fetus, there is no universal fetal marker; in a female fetus measurement is more challenging. We have developed a panel of multiplexed insertion/deletion polymorphisms that can measure fetal fraction in all pregnancies in a simple, targeted sequencing reaction.

**Methods:**

A multiplex panel of primers was designed for 35 indels plus a *ZFX/ZFY* amplicon. cfDNA was extracted from plasma from 157 pregnant women, and maternal genomic DNA was extracted for 20 of these samples for panel validation. Sixty-one samples from pregnancies with a male fetus were subjected to whole genome sequencing on the Ion Proton sequencing platform, and fetal fraction derived from Y chromosome counts was compared to fetal fraction measured using the indel panel. A total of 157 cell-free DNA samples were sequenced using the indel panel, and informativity was assessed, along with the proportion of fetal DNA.

**Results:**

Using gDNA we optimised the indel panel, removing amplicons giving rise to PCR bias. Good correlation was found between fetal fraction using indels and using whole genome sequencing of the Y chromosome (Spearmans r = 0.69). A median of 12 indels were informative per sample. The indel panel was informative in 157/157 cases (mean fetal fraction 14.4% (±0.58%)).

**Conclusions:**

Using our targeted next generation sequencing panel we can readily assess the fetal DNA percentage in male and female pregnancies.

## Introduction

Traditional methods of prenatal diagnosis rely upon invasive procedures including amniocentesis and chorionic villus sampling (CVS), which are associated with a small risk of miscarriage [1]. The discovery of cffDNA in maternal plasma [2] has allowed development of non-invasive prenatal diagnosis for single gene disorders [3], as well as non-invasive prenatal testing (NIPT) for aneuploidy [4–8]. cffDNA can also be used as a biomarker to monitor high-risk pregnancies, for example those at risk of pre-eclampsia [9], pre-term labour [10], or fetal maternal haemorrhage [11]. The majority of the cfDNA obtained from plasma is maternal; fetal DNA has been shown to constitute around 10–15% [5,12], but can vary from less than 4% to greater than 30% [13]. Factors influencing the percentage of cffDNA detected include maternal weight [6], placental health [9,10], time from blood-draw to processing [14] and blood storage factors [15,16]. A fetal fraction below 4% occurs in only 1–3% of pregnancies [6].

NIPT using cfDNA and massively parallel sequencing relies on looking for an over- or under-representation of an entire chromosome or region of interest (for example, chromosome 21) compared to the same chromosome or region in euploid samples. The higher the percentage of cffDNA present, the easier it is to detect these differences [13]. Commercial companies each have a threshold below which they consider NIPT results to be unreliable, usually 3.5–4% [6,17]. Recent work has suggested that it is essential to assess fetal fraction when reporting NIPT results, since at very low fetal fraction a ‘normal’ result may be obtained, with potential to be a false negative [18]. Knowledge of the fetal fraction is also essential for carrying out tests for single gene disorders using techniques such as relative mutation dosage (RMD) and relative haplotype dosage, where it is necessary for calculating whether a mutant allele is over- or under-represented compared to the wild-type allele [19–22]. Again, a higher percentage of cffDNA makes detection of an over-represented allele easier.

Measurement is straightforward in the case of a male fetus, since Y-chromosome markers are readily detectable [2,12]. However, in the case of a female fetus, there is no universal fetal marker, thus quantification is more challenging. A number of different approaches using massively parallel sequencing have been shown to be effective for assessing fetal fraction as part of the NIPT work flow [23–27]. Although these whole-genome sequencing methods are accurate, they are costly, and require complicated bioinformatics algorithms. Assays using markers that are hypermethylated in the placenta and hypomethylated in the maternal genomic DNA, such as the *SOX14*, *TBX3*, and *RASSF1A* markers [28, 29], have been developed. However, these approaches may not be ideal, since it has been shown that global methylation levels change during pregnancy [30], and it is possible that methylation levels of some markers may not be stable. Digital PCR using probes specific for indels has been used to accurately quantify fetal fraction [20]; however, the panel of polymorphisms examined was only informative in 65% of female-bearing pregnancies, indicating that a larger number of polymorphisms should be examined to allow quantification in all pregnancies. Multiplexed digital PCR to increase the panel size is possible [31,32], but the throughput is still relatively low, with five targets reported in a single reaction by Zhong *et al*. [31]. In order to screen a large number of polymorphisms per sample, multiple reactions would be required using this method.

Here we describe a simple amplicon-based sequencing approach to quantify fetal fraction using a panel of 35 insertion/deletion polymorphism markers. This panel can be easily incorporated into lab workflows for single gene disorders without adversely affecting the number of reads for the samples. The panel was informative in all samples tested.

## Methods

### Patient recruitment

10 mL of blood was collected into K_3_-EDTA tubes from one non-pregnant female and one male for validation of PCR primers. 10–20 mL blood was collected from 157 women attending National University Hospital, Singapore, for routine antenatal appointments. Median gestational age was 18+3 weeks (IQR: 12+3 to 24+0 weeks). Informed consent was obtained in writing prior to venipuncture and the study was approved by the National Healthcare Group Domain Specific Review Board, Singapore (DSRB2013/00837). Investigations were conducted according to the principles expressed in the Declaration of Helsinki.

### Sample processing

Plasma was processed and stored as described previously [20]. Time from blood draw to processing was less than four hours in all cases.

### DNA extraction

Cell-free DNA was extracted from plasma using the QiaAmp Circulating Nucleic Acid kit (Qiagen) and was eluted into 75 μL of elution buffer. 4 mL of plasma was used for male samples comparing the three methods of fetal fraction calculation, and 2 mL of plasma was used for samples used for all other indel assays. Genomic DNA was extracted from 250 μL of blood using an E.Z.N.A Blood DNA Mini kit (Simply Science), and was eluted into 100 μL elution buffer.

### Serially diluted model mixture preparation

Fetal gDNA from an amniocentesis sample and matched maternal gDNA were sonicated using a Bioruptor (Diagenode) with 30 cycles of 30 seconds on, 90 seconds off, on high power to give DNA fragments of approximately 200 bp, representing a similar size to cfDNA fragments. Fetal gDNA was then diluted in maternal genomic DNA to give two-fold serial dilution from 50% fetal DNA down to 1.6%.

### Indel panel design

Non-coding bi-allelic indels with a global minor allele frequency of >0.25 and/or an average heterozygosity of >0.3, and an allele length variation of 2–10 base pairs (bp) were selected from the Marshfield database [33]. Flanking sequences of these indels, as well as reported sequence variants within this region, were obtained using the University of California Santa Cruz Genome Browser [34] (Human hg19 database, Genome Reference Consortium GRCh37, release date Feb 2009) at http://genome.ucsc.edu/. Primer design was performed using Primer3 software [35]. We selected amplicons of between 68–120 bp in length with annealing temperatures of 58–62°C (S1 Table). Primer pairs were checked for specificity using the National Centre for Biotechnology Information Basic Local Alignment Search Tool (BLAST; http://www.ncbi.nlm.nih.gov/tools/primer-blast/). We used Multiple Primer Analyzer to check for the presence of homo- and heterodimers and hairpins (http://www.thermoscientificbio.com/webtools/multipleprimer/). A total of 44 biallelic indels spread across all human autosomes, excluding chromosomes 20 and 21, were pooled into three PCR multiplex mixes.

### Indel sequencing library prep

PCRs were carried out using 12.5 μL of 2x Quantifast Multiplex PCR Mix (Qiagen), 200 nmol/L each primer, and 4.5 μL plasma DNA or gDNA (1 ng/μL), in a final volume of 25 μL. Cycling conditions were as follows: 10 mins 95°C, then 25 cycles of 30s at 95°C, 90s at 60°C and 90s at 72°C with a final extension of 72°C for 10 minutes. Libraries were prepared using TruSeq Nano DNA Sample Preparation kits (Illumina) with indexed adaptors diluted 1:100.

To determine whether the number of read counts per sample was affected by the choice of sequencing library preparation kit, we amplified cfDNA from six patients carrying a male fetus using the indel panel, split the PCR products into three and prepared indexed libraries for each using three library prep kits: PCR-free DNA Sample Preparation kit (Illumina), TruSeq Nano DNA Sample Preparation kit, and ThruPLEX-FD Preparation kit (Rubicon Genomics).

Purified libraries were diluted to 4 nM and equal amounts of 24 samples were pooled to yield a single 4 nM library. The library was diluted to a final concentration of 10 pM, loaded into MiSeq v3 150 cycle cartridges and 100 cycles of single-end sequencing was initiated.

### Indel data analysis

A detailed protocol for data analysis is given in S1 Methods. Briefly, fastq files were analysed using a Python script to count forward and reverse reads generated for every allele, using a few bases up-stream and down-stream of the indel sequence and including the indel sequence itself, as the search sequences. Fetal fraction was calculated as follows:

f=((2×fetalallele)÷(sharedallele+fetalallele))×100%

The mean fetal fraction given all informative indels for each sample was used as an estimate of the fetal fraction. Indels yielding giving a fetal fraction lower than 1.5% were excluded from analysis.

### Library preparation and data analysis for whole genome sequencing (WGS)

Libraries for WGS were prepared using the Ion Plus Fragment Library Kit (Thermo Fisher Scientific) according to manufacturer’s instructions, with addition of indexes for each sample. Samples were diluted to 100 pM and pooled in batches of ten. 45 pM of pooled DNA was loaded onto the Ion One Touch 2 System (Thermo Fisher Scientific), and library amplification and enrichment were carried out according to manufacturer’s instructions. Sequencing was performed on the Ion Proton (Thermo Fisher Scientific) using a v3 chip. Duplicate reads were removed using Picard, and data was analysed using the RAPIDR analysis package in R [36]. Fetal fraction for male samples is included in the output from this package.

### Statistical analysis

A Friedman test followed by Dunn’s multiple comparisons test was used to compare the performance of the three sequencing kits. Wilcoxon signed rank tests were used to calculate all other statistical differences. Test were two-tailed, and p<0.05 was considered significant. All statistical calculations were performed using GraphPad Prism 7.0 (GraphPad Prism).

## Results

### Optimisation of multiplex indel sequencing assay

PCR primers for each indel amplicon tested on a non-pregnant female control gDNA sample produced single bands of the correct size on agarose gel electrophoresis. Primers were multiplexed and used to amplify the indels on gDNAs from a normal non-pregnant female control and a male control gDNA sample indicated that all indels were amplified successfully in 15-plex pools. To look for PCR bias, we extracted gDNA from maternal blood from 20 pregnant women, amplified the DNAs using the panel of 44 indels plus the *ZFX/ZFY* marker, and sequenced. A total of 6,927,781 sequenced reads were generated for these 20 samples, giving a median read count per sample of 124,128 (IQR: 71,366–217,866; S1A Fig). The median number of reads per indel (S1B Fig) ranged from 701 (MID1823) to 27,234 (MID187). The ratio of counts of allele A to allele B is expected to be 0.0 if the sample is homozygous for either allele (using the allele with the larger read count as the denominator), and will be 1.0 if the woman is heterozygous at a particular locus. We excluded any amplicons that were homozygous but had a ratio of greater than 0.01 or were heterozygous with a ratio of less than 0.9. We also excluded indels that yielded a mean of less than 2,000 reads; we thus excluded a total of 9 indels from our panel (S2 Table). The remaining indels were deemed suitable for accurate and non-biased amplification, resulting in a panel of thirty-five indels plus the *ZFX/ZFY* amplicon. *ZFX/ZFY* was expected to give a ratio of 0.0 in all cases, since the gDNAs are from female patients, and this was observed to be true.

We compared three library prep kits using six samples, and found that there was a significantly higher mean total number of reads generated using the TruSeq PCR-free kit compared to the ThruPLEX-FD kit (p = 0.03; S2A Fig), but no significant difference in the number of informative indels between any of the kits (*p* = 0.74, S2B Fig, S3 Table). There was no significant difference between the estimated fetal fractions for each sample using any of the three kits (S2C Fig, S3 Table). We opted to continue using the TruSeq Nano kit for our analyses.

### Correlation of observed versus expected fetal fraction using a model mixture

**Fig 1:**
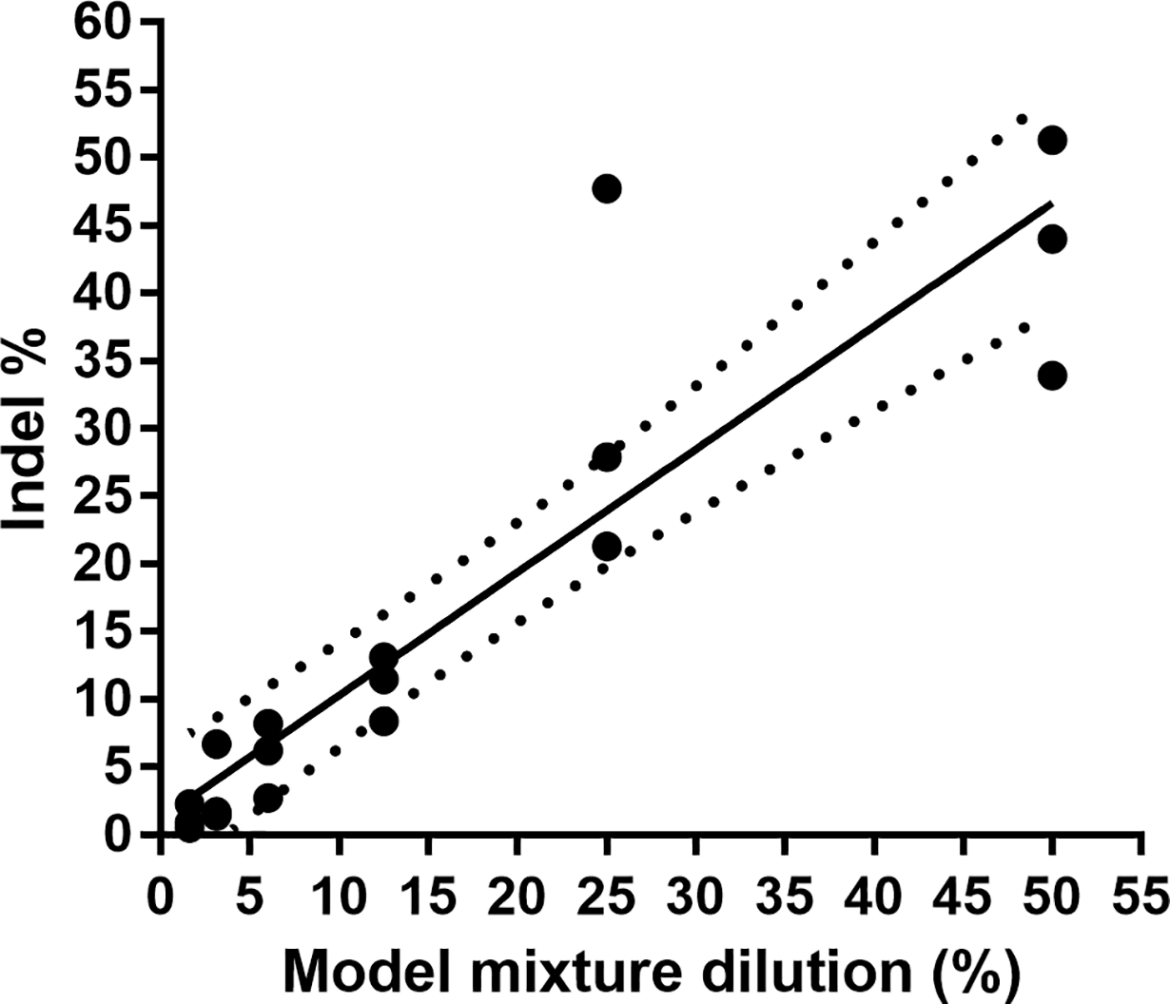
Linear regression of measurement of fetal fraction in a serially diluted model mixture using the panel of indels. Measurements by three informative indels are shown for each dilution point. Slope = 0.91 (0.69–1.12), r^2^ = 0.83, p<0.0001. The dotted lines represent the 95% confidence interval of the line of best fit.

Genomic DNA was extracted from both maternal blood cells and amniotic fluid from a patient carrying a male fetus, and both samples were sequenced using the indel panel. Concurrently, a serially diluted model mixture containing 50% to 1.6% of fetal gDNA diluted in maternal gDNA was also sequenced. Three informative indels, absent in the mother and present in the cfDNA, were identified and used to estimate fetal fractions in the dilution series. We found that the assay was quantitative down to 1.6% fetal DNA (SEM±0.55%) (Table 1). A strong potential for the indels to accurately predict the fetal fraction was found using linear regression (Fig 1). In contrast, fetal fraction estimated for each dilution using the Y-chromosome sequence *ZFY* as a ratio of the homologous *ZFX* sequence did not estimate fetal fraction accurately (slope = 0.22 (0.08–0.36), r^2^ = 0.83, *p = 0*.012; S3 Fig).

**Table 1:** Model mixture used to evaluate the limit of sensitivity for the indel panel. Three informative indels were found and it was shown that the assay is sensitive down to 1.6% fetal DNA.

					1.6%		3.1%		6.3%		12.5%		25.0%		50.0%	
Indel	Counts mat gDNA	Ratio mat alleles	Counts AF	Ratio AF alleles	Counts	Indel %	Counts	Indel %	Counts	Indel %	Counts	Indel %	Counts	Indel %	Counts	Indel %
MID1372_A	3,132	0.02	1,308	0.91	1,909	2.3	551	6.7	1,361	8.2	1,456	8.4	268	47.7	1,866	44.0
MID1372_B	63		1,442		22		20		58		64		84		527	
MID1782_A	11,192	0.00	8,672	0.90	1,277	0.5	442	1.4	1,610	2.7	1,660	11.5	271	27.9	1,656	51.3
MID1782_B	6		9,681		3		3		22		101		44		572	
MID1384_A	12,155	0.00	8,051	0.96	1605	0.9	355	1.7	1,032	6.2	1,280	13.1	519	21.3	1,694	33.9
MID1384_B	6		8,363		7		3		33		90		62		346	
**Percentage indel (±SEM)**	** **				**1.0% (+/- 0.55)**		**2.5% (+/- 1.72)**		**5.2% (+/-1.6)**		**10.8% (+/- 1.38)**		**30.5% (+/- 7.93)**		**42.5% (+/- 5.05)**	

AF = amniotic fluid

### Validation of the indel assay using Y-chromosome sequences

**Fig 2:**
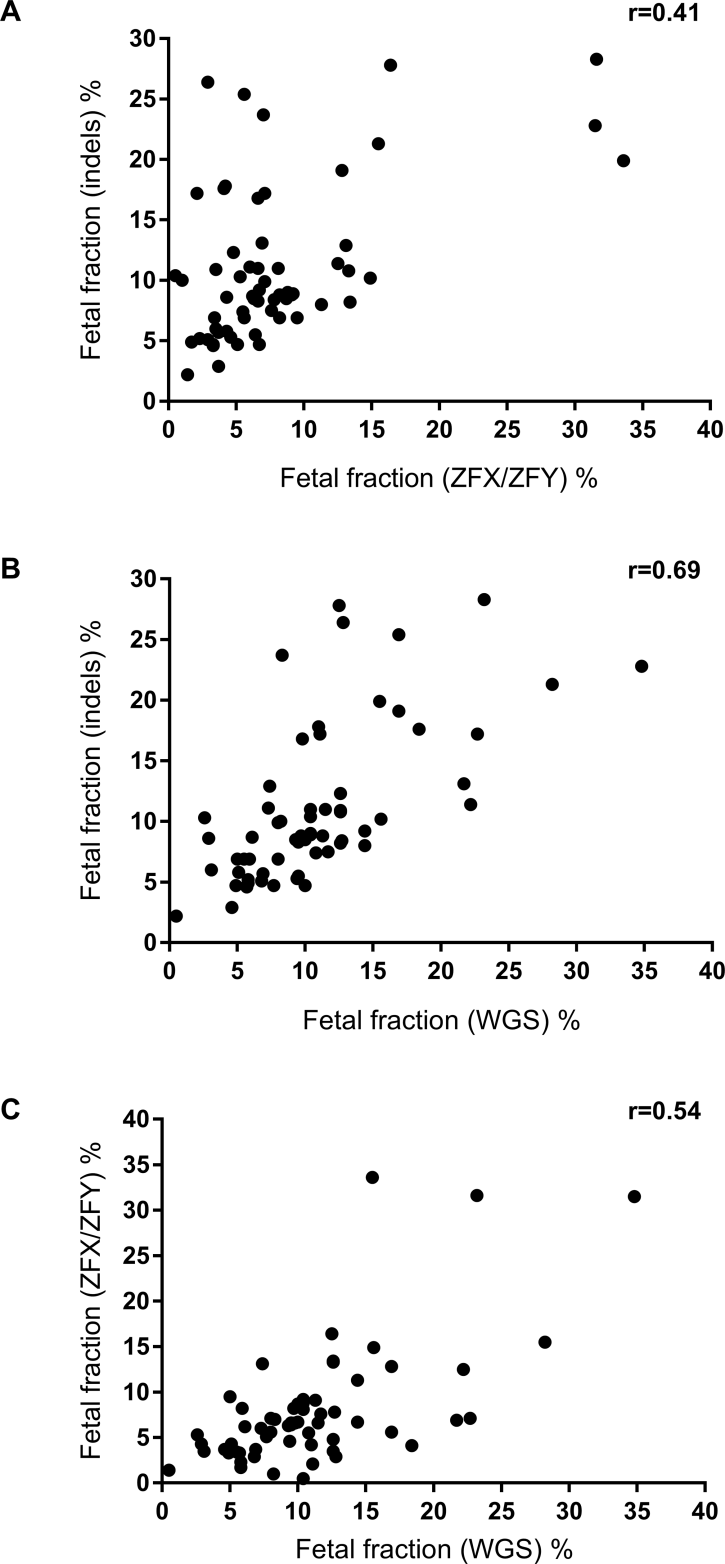
Measurement of fetal fraction using Y chromosome sequences. Correlation of fetal fraction measured by (A) *ZFX/ZFY* and the indels, (B) indels and WGS, and (C) WGS and *ZFX/ZFY*.

Fetal fraction measurements using the indel panel were compared to measurements using the *ZFX/ZFY* markers in 61 samples from pregnancies with a male fetus. There was a significant difference in the mean fetal fractions obtained using the indels and *ZFX/ZFY* (11.1% vs 7.9%; *p*<0.0001; S4 Fig). Correlation was found between the fetal fraction estimated using the two assays (Spearman r = 0.41 (95% CI: 0.17–0.61), *p* = 0.0009; Fig 2A, S4 Table). Since the *ZFX/ZFY* assay is based upon detection of just a single amplicon, which may be less reliable for sequencing than using a panel of amplicons, the fetal fraction using the indel panel measured on the MiSeq was also compared to fetal fraction estimated using WGS read counts from the entire Y chromosome, measured on the Ion Proton sequencing platform, using the same 61 samples. WGS should give the most accurate estimation of the fetal fraction due to the high number of data points. The mean fetal fraction estimate using the indel panel was the same as that using WGS (11.1% vs 11.0%, p = 0.73, S4 Fig), and a high degree of correlation was found (Fig 2B, S3 Table; Spearman r = 0.69 (95% CI: 0.53–0.81), *p*<0.0001). Correlation of the ZFX/ZFY assay with WGS assay gave a Spearman r value of 0.54 (95% CI: 0.33–0.70), indicating that single amplicon does not give such an accurate estimation of fetal fraction as the indel panel (Fig 2C, S4 Table).

### Informativity of the indel panel

**Fig 3:**
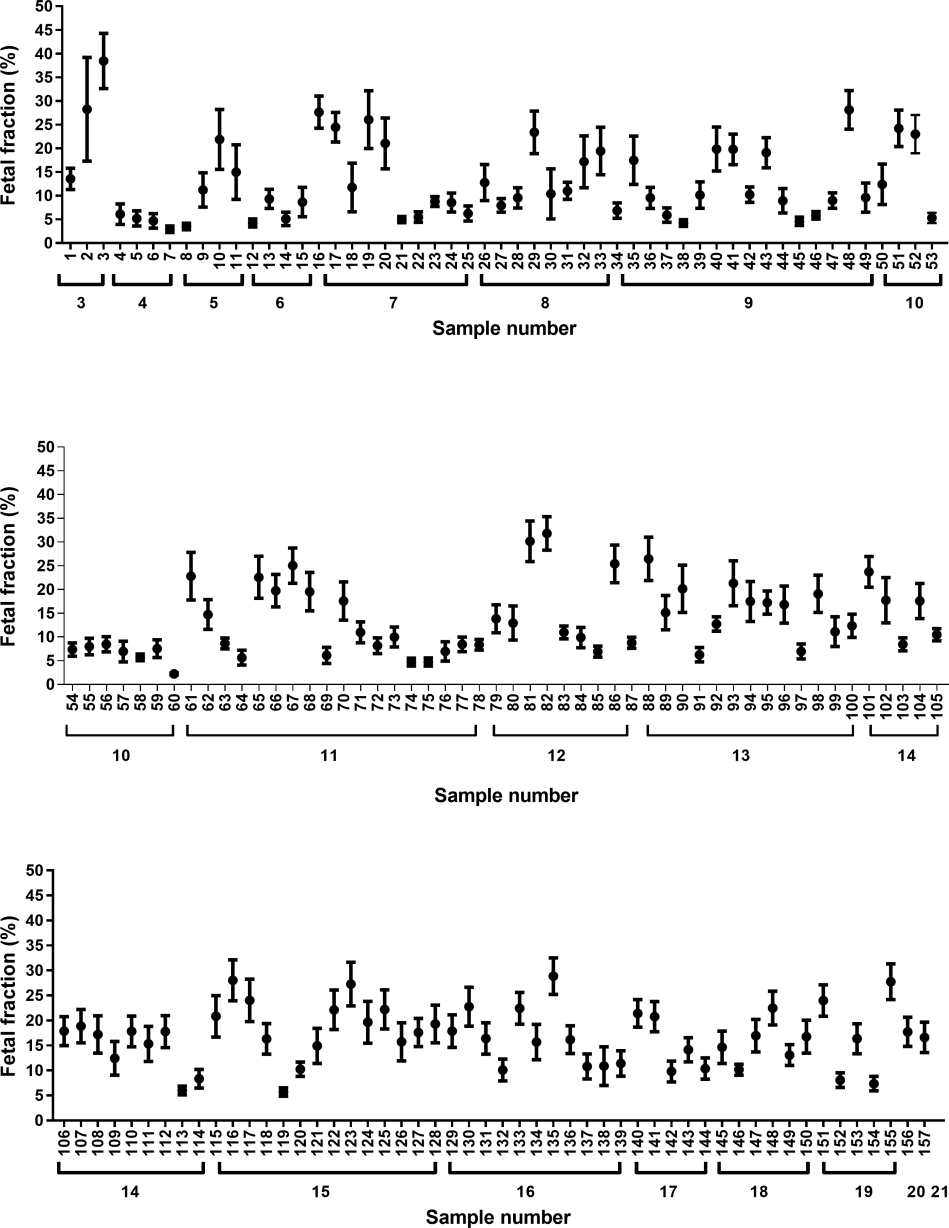
Estimated fetal fraction for 157 cfDNA samples. The black circles represent the geometric mean of each sample, and the 95% confidence intervals of the geometric mean are indicated with horizontal bars. The numbers below the X-axis indicate the number of informative indels for each sample.

Following validation, the indel panel was used to estimate the fetal fraction in samples from a further 90 pregnant women, giving data for a total of 157 samples (Fig 3, S5 Table; detailed coverage of the fetal fractions for each indel for each sample shown in S6 Table). The number of informative indels per sample was recorded (S5 Table), showing a median of 12 indels per sample (IQR: 9–15). The minimum number of informative indels per patient was three (n = 3), and the maximum number of informative indels was twenty-one (n = 1). We recorded patient ethnicity (Chinese, Malay, Indian, and Other) for all patients, and did not observe any apparent bias in the number of informative indels for any ethnic group (S5 Fig). The percentage informativity for each indel ranged from 15.3% to 45.9% (S7 Table). The mean fetal fraction measured was 14.4% (SEM±0.58%).

## Discussion

We have developed a simple assay to measure fetal fraction, applicable to both male and female pregnancies, using a panel of 35 indel markers. Quantification using the indel panel correlates well with values obtained using whole genome next generation sequencing to measure fetal fraction using the Y chromosome, regarded as the current ‘gold standard’ for fetal fraction measurement [37]. We have shown that the panel is informative in 100% of cases tested to date (157/157), therefore should be applicable to most pregnancies.

Forty-four indels were initially selected to give three pools of 15 amplicons, including *ZFX/ZFY*. Many more indels were available for inclusion, but we wanted to balance finding as many indels as possible to maximize informativity and keeping the analysis simple. Examination of the raw data for 20 gDNA samples showed few false positive counts produced using the indel panel. An average cut-off ratio of 0.01 was applied for each homozygous allele, above which an indel was excluded; this led to exclusion of two indels. Four indels were also excluded due to a tendency to give a ratio of allele A to allele B of less than 0.9 in samples heterozygous for two alleles. The main source of sequencing errors for the MiSeq sequencing platform are substitution miscalls [38], due to similarities in emission spectra between the fluorophores used for A and C as well as between those used for G and T. We used indels rather than single nucleotide polymorphisms in our panel to prevent any false positives caused by substitution errors, since it would require two or more consecutive substitutions to occur; although still possible, the chances are extremely low. It is arguable that a potential cause of PCR bias is that alleles with larger deletions would be expected to amplify better than those without the deletion; however, we did not find this to be the case. MID187 has a ten bp difference in size between its two alleles, and still consistently gave an A:B ratio of close to 1.0. Conversely, one of the excluded indels (MID2592) had only a 2bp difference in size between its alleles and yet had a median A:B ratio of 0.86.

Using a model mixture, we demonstrated that the indel panel shows significant correlation over the titration range tested. It is quantitative down to 1.6% of fetal DNA, although the limit of quantification will need to be studied in a larger number of model mixtures to be more accurately determined. We chose to exclude fetal fractions below 1.5% from our analysis of cfDNA samples, since this will remove any potential ‘sequencing noise’ [39]. Whilst we were initially concerned that this may lead to an over-estimation of fetal fraction, since there may be indels with a real contribution of below 1.5%, comparison with the fetal fraction measured using WGS showed that in fact the mean fetal fraction using indels was the same as that using WGS (11.1% and 11.0% respectively). Inspection of the raw data informs us how many indels had to be excluded for each sample, and an unusually high number of low percentage alleles would indicate that a sample may have a fetal fraction below 1.5%.

Chromosome Y reads produced by whole genome sequencing approaches are frequently used to determine fetal fraction [5,37,40]. Methods including SANEFALCON [27], which makes use of nucleosome profiling to assess fetal fraction, and SEQFF [24], which uses a high dimensional regression model of existing NIPT sequencing data, can be used to assess fetal fraction in pregnancies with a fetus of either sex. FetalQuant^SD^ has been recently developed for fetal fraction quantification, based on shallow-depth WGS, using SNPs absent from the maternal genome at regions where the mother is homozygous for the alternative allele [41] However, all of these methods are based on whole genome sequencing; we wanted to develop a relatively inexpensive targeted amplicon-based approach which could be used in conjunction with our RMD assays for single-gene disorders, such as β-thalassaemia. Whole genome sequencing to assess the fetal fraction in these instances would be prohibitively expensive, whereas we can multiplex our targeted indel panel onto a run with the single-gene disorder samples with little impact on the number of reads that we obtain for the β-thalassaemia targets. We require a minimum of 2,000 reads per informative indel, and so conservatively would hope for a total of 72,000 reads per sample as a minimum (assuming that all indels plus the ZFX/ZFY amplicon were informative). Given that 25 million reads can be readily achieved using a MiSeq flow cell, it will be simple to add in the indels for samples as part of a routine MiSeq run without compromising the quality of data or number of samples that can be run for other assays.

In the event of a female fetus, there is no single reliable marker identified to date to estimate fetal fraction, but even if a single marker were to be identified, we believe it to be more accurate when sequencing to use multiple amplicons rather than relying on just one. This is borne out by our data showing that the ZFX/ZFY assays does not give such a robust correlation with WGS as the indel assay does. Recent work by Chan *et al*. [42] has suggested that cffDNA is fragmented at ‘preferred ends’ (specific cutting sites throughout the genome) and it is unknown, as yet, whether these ‘preferred ends’ are patient specific sites or whether they are applicable to the whole population. Similarly, nucleosome profiling on cfDNA performed by Straver *et al*. [26] suggested specific read start sites, which may indicate specific fragmentation sites. If a single amplicon has PCR primers falling on either side of a ‘preferred end’ fragmentation site, then it would be far less likely to amplify, leading to allele dropout, and would thus be less reliable than an amplicon where both primers sat between two cutting sites. Using multiple amplicons would reduce the chance of underestimation of fetal fraction due to allele dropout, and will allow for a fetal fraction to be calculated even if one particular allele cannot be amplified.

When a maternal blood sample is sent to different commercial providers, the estimate of fetal fraction can be quite variable, and the fetal fraction that they provide is just an estimate. There is no standardization of methods for assessment of fetal fraction [43], and so it is possible that we would have achieved a better correlation of indels with Y chromosome sequencing using a different algorithm. Achieving a definitive ‘correct’ fetal fraction will be difficult. The correlation of the indel panel with the Y chromosome sequencing was 0.69. This is comparable to the correlations found in recently published studies by two other groups (0.65 and 0.66, refs [26,40] respectively), but it should be noted that the indel panel requires additional sequencing to obtain the fetal fraction, whereas fetal fraction using whole Y chromosome data was obtained without additional sequencing. Therefore, the indel panel does not provide an advance over these two methods for assessing fetal fraction in cases where whole genome sequencing will later be performed (for example, for NIPT). However, it is still preferable to use the indel panel, with a reasonable (albeit lower) correlation, when performing targeted sequencing in order to keep assay costs down.

There is the possibility that for some patients, the panel may lack informativity, for example in the case of consanguineous marriage. The indels were selected from a database with heterozygosity information for European, Japanese, African and Native American populations [32], and in our cohort of patients living in Singapore, from a variety of ethnic backgrounds, all patients were informative for at least three indels. The danger of relying on a low number of indels is that the measurement could be overly affected by a single outlying value (for example, our sample 2, with only three informative indels, had a high SEM). It will be necessary to exercise caution in these samples with a low number of informative indels.

As well as being applicable to relative mutation dosage, a number of studies have shown that cffDNA can be used to monitor at-risk pregnancies [9–12,44]. Our assay could be used in these cases by comparing maternal gDNA and a corresponding cfDNA sample at the start of the pregnancy to establish which indels are informative, then assaying samples collected at regular intervals using primers specific for the informative indels only. It may also be possible to use the panel for monitoring transplant patients for organ rejection, since it has been shown that the donor DNA is present in recipient cfDNA [45]. Donor DNA levels are predicted to increase during rejection, and an assay using SNPs to monitor cardiac transplant patients has already been reported [46]. However, the methods used for this study required microarray and whole genome sequencing analysis, which are both costly and time-consuming compared to our method.

In conclusion, we feel that the simplicity of the assay, ease of analysis, and sensitivity of the test indicate that our indel panel can be easily implemented in any laboratory with a bench top sequencer, with potential for many applications.

## Supporting information

S1 Methods Analysis of data generated using the indel panel. (DOCX)

S1 Table Insertion/deletion polymorphisms and corresponding primer sequences. (DOCX)

S2 Table Ratios of allele A:allele B for each indel marker. Twenty gDNA samples were sequenced and the number of homozygous and heterozygous markers was recorded. The ratio of allele A:B was calculated. Any indel with a median of greater than 0.01 for a homozygous indel or less than 0.9 for a heterozygous indel was excluded from further analysis. Additionally, indels yielding a median of fewer than 2,000 reads were also excluded. Excluded indels are shaded in grey.
(DOCX)

S3 Table Comparison of fetal fraction obtained using three different library preparation kits. Figures in brackets show the number of informative indels for each sample.
(DOCX)

S4 Table Fetal fractions for 61 samples estimated using WGS, the indel panel, and the *ZFX/ZFY* amplicon. (DOCX)

S5 Table Patient demographics and sequencing data for 157 samples tested using the indel panel to quantify fetal fraction. ‘-‘ indicates that the data was not collected. Samples highlighted in grey were also used for the comparison of methods used for measuring fetal fraction. Samples with ‘*’ were used to compare the library prep kits.
(DOCX)

S6 Table The detailed coverage of the indels for 157 samples tested. The fetal fraction for each informative indel is listed. Estimated fetal fraction is calculated, and the number of informative indels per sample is also shown.
(XLSX)

S7 Table Percentage of samples in which each indel is informative. Total sample number, n = 157.
(DOCX)

S1 Fig Optimisation of the indel panel using gDNA. (A) Number of reads generated using the indel panel for each gDNA sample. The median and interquartile range are indicated; (B) A Tukey box plot showing median number of reads per indel amplicon. Median and interquartile range are shown by grey boxes, median is shown by the horizontal line, and whiskers represent the range of the data. Outliers are indicated by black circles.
(TIF)

S2 Fig Comparison of different library prep kits on estimate of fetal fraction. Three kits were compared, namely the PCR-free DNA Sample Preparation kit, the TruSeq Nano DNA Sample Preparation kit, and the ThruPLEX kit. A) There is a significantly higher number of reads from the PCR-free kit compared to Thruplex; B) No difference was seen between the kits in number of informative indels; C) No difference was seen in the fetal fraction for any of the six samples between the three kits. Each dot represents an individual indel. Horizontal bars represent the mean estimation of fetal fraction, and the standard error of the mean (SEM) is shown for each sample.
(TIF)

S3 Fig Linear regression of model mixture dilutions for fetal DNA versus fetal fraction measured using a single *ZFX/ZFY* amplicon to detect Y chromosome sequences. Slope = 0.22 (0.08–0.36), r^2^ = 0.83, *p = 0*.012. The 95% confidence intervals of the slope are represented by dotted lines.
(TIF)

S4 Fig Scatter plots showing mean and SEM for the fetal fractions measured for 61 samples from male-bearing pregnancies using WGS, the indel panel and the *ZFX/ZFY* assay for the Y chromosome to measure fetal fraction. (TIF)

S5 Fig Number of informative indels per patient. Ethnicity for all 157 patients was recorded: Chinese, n = 76; Malay, n = 13; Indian, n = 33; Others, n = 35.
(TIF)
