# Supplementary material for: Measurement of fetal fraction in cell-free DNA from maternal plasma using a panel of insertion/deletion polymorphisms
Source: PLoS One. 2017 Oct 30;12(10):e0186771. doi: 10.1371/journal.pone.0186771 (PMC5662091; doi:10.1371/journal.pone.0186771)
Supplement: S1 Methods — (DOCX) [file pone.0186771.s001.docx]

**Determining fetal fraction using a panel of insertion/deletion polymorphisms**

Fastq files were analysed using a Python script to count forward and reverse reads generated for every allele. This script searched for the sequence of the DNA region containing the insertion (plus/minus a few bases), as well as searching for the sequence without the insertion (plus/minus a few bases).

For example, the search sequences for MID187 (rs16626) are as follows (the indel region is highlighted in bold):

Allele A Forward: ACCC**CGTGAAGTCC**TAT

Allele A Reverse: ATA**GGACTTCAC**GGGGT

Allele B Forward: ACCCTATGGGTTGGGAC

Allele B Reverse: GTCCCAACCCATAGGGT

The forward and reverse reads were summed for each allele, and then the fetal fraction for each indel was calculated using the following formula:

f = (2 x fetal allele / (shared allele + fetal allele)) x 100%.

An indel was classed as ‘informative’ if it gave a fetal fraction above 1.5% but below 50%. The mean fetal fraction for all informative indels for each sample was used as an estimate of the final fetal fraction. We did not include the ZFX/ZFY fetal fraction in the calculation for mean fetal fraction, since it is not applicable in female pregnancies.

For a worked example, see below:

**Sample 31**

|  | **Sample 31** | **Sum (Forward + Reverse)** | **Fetal Fraction (%)** |
| --- | --- | --- | --- |
| MID187_A | 5 | 10 |  |
| MID187_A_R | 5 |  |  |
| MID187_B | 19503 | 37010 |  |
| MID187_B_R | 17507 |  |  |
| MID2050_A | 9089 | 17861 | 7.1 |
| MID2050_A_R | 8772 |  |  |
| MID2050_B | 360 | 653 |  |
| MID2050_B_R | 293 |  |  |
| MID2045_A | 12280 | 23998 | 12.4 |
| MID2045_A_R | 11718 |  |  |
| MID2045_B | 820 | 1585 |  |
| MID2045_B_R | 765 |  |  |
| MID1372_A | 48 | 86 |  |
| MID1372_A_R | 38 |  |  |
| MID1372_B | 6602 | 13358 |  |
| MID1372_B_R | 6756 |  |  |
| MID1830_A | 2869 | 5119 |  |
| MID1830_A_R | 2250 |  |  |
| MID1830_B | 2288 | 4430 |  |
| MID1830_B_R | 2142 |  |  |
| MID785_A | 1973 | 4144 | 7.7 |
| MID785_A_R | 2171 |  |  |
| MID785_B | 77 | 165 |  |
| MID785_B_R | 88 |  |  |
| MID1514_A | 18465 | 35705 | 7.2 |
| MID1514_A_R | 17240 |  |  |
| MID1514_B | 891 | 1327 |  |
| MID1514_B_R | 436 |  |  |
| MID1643_A | 10027 | 19729 |  |
| MID1643_A_R | 9702 |  |  |
| MID1643_B | 8689 | 17311 |  |
| MID1643_B_R | 8622 |  |  |
| MID1945_A | 2035 | 4295 |  |
| MID1945_A_R | 2260 |  |  |
| MID1945_B | 3235 | 6761 |  |
| MID1945_B_R | 3526 |  |  |
| MID1782_A | 14396 | 29303 |  |
| MID1782_A_R | 14907 |  |  |
| MID1782_B | 2 | 3 |  |
| MID1782_B_R | 1 |  |  |
| MID3031_A | 6206 | 12105 | 13.3 |
| MID3031_A_R | 5899 |  |  |
| MID3031_B | 444 | 861 |  |
| MID3031_B_R | 417 |  |  |
| MID116_A | 8060 | 16816 |  |
| MID116_A_R | 8756 |  |  |
| MID116_B | 6 | 9 |  |
| MID116_B_R | 3 |  |  |
| MID1209_A | 466 | 1003 |  |
| MID1209_A_R | 537 |  |  |
| MID1209_B | 204 | 420 |  |
| MID1209_B_R | 216 |  |  |
| MID1384_A | 3 | 4 |  |
| MID1384_A_R | 1 |  |  |
| MID1384_B | 1071 | 2190 |  |
| MID1384_B_R | 1119 |  |  |
| MID520_A | 2951 | 5692 |  |
| MID520_A_R | 2741 |  |  |
| MID520_B | 1463 | 2913 |  |
| MID520_B_R | 1450 |  |  |
| MID649_A | 706 | 1436 |  |
| MID649_A_R | 730 |  |  |
| MID649_B | 386 | 796 |  |
| MID649_B_R | 410 |  |  |
| MID3220_A | 6603 | 13999 |  |
| MID3220_A_R | 7396 |  |  |
| MID3220_B | 5663 | 11958 |  |
| MID3220_B_R | 6295 |  |  |
| MID3321_A | 23871 | 46923 |  |
| MID3321_A_R | 23052 |  |  |
| MID3321_B | 52 | 94 |  |
| MID3321_B_R | 42 |  |  |
| MID1522_A | 3556 | 7028 | 9.5 |
| MID1522_A_R | 3472 |  |  |
| MID1522_B | 163 | 352 |  |
| MID1522_B_R | 189 |  |  |
| MID257_A | 8072 | 15705 |  |
| MID257_A_R | 7633 |  |  |
| MID257_B | 5 | 9 |  |
| MID257_B_R | 4 |  |  |
| MID1997_A | 19498 | 38152 |  |
| MID1997_A_R | 18654 |  |  |
| MID1997_B | 1 | 8 |  |
| MID1997_B_R | 7 |  |  |
| MID1824_A | 49 | 103 |  |
| MID1824_A_R | 54 |  |  |
| MID1824_B | 239 | 284 |  |
| MID1824_B_R | 45 |  |  |
| MID1120_A | 4405 | 8841 |  |
| MID1120_A_R | 4436 |  |  |
| MID1120_B | 4 | 8 |  |
| MID1120_B_R | 4 |  |  |
| MID2057_A | 331 | 698 |  |
| MID2057_A_R | 367 |  |  |
| MID2057_B | 309 | 609 |  |
| MID2057_B_R | 300 |  |  |
| MID2648_A | 986 | 1954 |  |
| MID2648_A_R | 968 |  |  |
| MID2648_B | 6 | 7 |  |
| MID2648_B_R | 1 |  |  |
| MID1436_A | 8187 | 16228 |  |
| MID1436_A_R | 8041 |  |  |
| MID1436_B | 5470 | 10920 |  |
| MID1436_B_R | 5450 |  |  |
| MID3097_A | 9835 | 19753 | 8.7 |
| MID3097_A_R | 9918 |  |  |
| MID3097_B | 432 | 902 |  |
| MID3097_B_R | 470 |  |  |
| MID1323_A | 8 | 20 |  |
| MID1323_A_R | 12 |  |  |
| MID1323_B | 51 | 94 |  |
| MID1323_B_R | 43 |  |  |
| MID1900_A | 191 | 658 |  |
| MID1900_A_R | 467 |  |  |
| MID1900_B | 0 | 2 |  |
| MID1900_B_R | 2 |  |  |
| MID2047_A | 10425 | 20360 |  |
| MID2047_A_R | 9935 |  |  |
| MID2047_B | 12640 | 24765 |  |
| MID2047_B_R | 12125 |  |  |
| MID1459_A | 1381 | 2762 | 22.3 |
| MID1459_A | 1381 |  |  |
| MID1459_B | 10969 | 22008 |  |
| MID1459_B_R | 11039 |  |  |
| MID770_A | 658 | 1331 |  |
| MID770_A_R | 673 |  |  |
| MID770_B | 319 | 665 |  |
| MID770_B_R | 346 |  |  |
| MID768_A | 64 | 107 |  |
| MID768_A_R | 43 |  |  |
| MID768_B | 287 | 541 |  |
| MID768_B_R | 254 |  |  |
| MID1561_A | 981 | 1988 |  |
| MID1561_A_R | 1007 |  |  |
| MID1561_B | 592 | 1213 |  |
| MID1561_B_R | 621 |  |  |
| MID1375_A | 1943 | 3983 |  |
| MID1375_A_R | 2040 |  |  |
| MID1375_B | 1510 | 3206 |  |
| MID1375_B_R | 1696 |  |  |
| ZFX | 3007 | 5965 | 6.6 |
| ZFX_R | 2958 |  |  |
| ZFY | 96 | 202 |  |
| ZFY_R | 106 |  |  |
| **Total_Reads** | 551503 |  |  |
| **Mean Fetal fraction (%)** |  |  | **11.0** |
