# Supplementary material for: Measurement of fetal fraction in cell-free DNA from maternal plasma using a panel of insertion/deletion polymorphisms
Source: PLoS One. 2017 Oct 30;12(10):e0186771. doi: 10.1371/journal.pone.0186771 (PMC5662091; doi:10.1371/journal.pone.0186771)
Supplement: S1 Table — (DOCX) [file pone.0186771.s002.docx]

| **Indel name** | **RefSNP name** | **Location** | **Indel** | **Amplicon size (min/max bp)** | **Forward Primer (5’>3’)** | **Reverse Primer (5’>3’)** |
| --- | --- | --- | --- | --- | --- | --- |
| MID187 | rs16626 | 1p34.1 | CGTGAAGTCC | 110/120 | ATATTCACCTCCAGCCACCA | CCATCTTCCCTACCAGACCA |
| MID2305 | rs3047269 | 1q23.3 | CTGA | 88/92 | CACACCAGCATGCAAGTACA | ATGGCCCATGAGAAACAAAC |
| MID1561 | rs397833115 | 2p24.3 | CTTC | 79/83 | ATAAAGGGCTCAGGGAGGAG | CCTGAAAGACCAAAATGTAAAACA |
| MID668 | rs71909697 | 2q22.3 | TACAA | 105/111 | CTGCAAAGGCAAAGCAAAAC | AGAGGACGGGACTTTCCAAT |
| MID185 | rs397821734 | 2q37.1 | GT | 116/118 | TTGACCAACATGTCCCCTTT | GCTGCACTGTTTTGTGAAAAAT |
| MID2050 | rs2308242 | 3p26.1 | CT | 116/118 | GAGCATGTTCTTCCCTCCAG | GCTCTGCGGAGTTCATTTTC |
| MID2045 | rs2308237 | 3q28 | TA | 110/112 | TTTAGGAACTAAGAAATGCATATAGGA | CAAACCTACTTTGCATTAGACACAA |
| MID1372 | rs150937808 | 4q21.1 | TTC | 103/106 | GCAGTTGACGAAGAACACGA | CCATGGGAAAAATGGATGAA |
| MID1830 | rs2308026 | 4q26 | CA | 103/105 | AAGCTGCAGGAATCACCAAG | ATGGCCCGTAGTCATCAATC |
| MID743 | rs1160956 | 5q12.3 | AGA | 82/85 | TTCTTTTCTAACAAGGAAAAGTTTGA | TCAAATTGTGTTCCTCCAAGG |
| MID768 | rs1160979 | 5q31.1 | AAG | 71/75 | ACCAGTAGAGTGGGGAGCAC | CCCACTATGCCCTACTGGAT |
| MID770 | rs1160981 | 5q31.1 | CAG | 86/89 | CTTCAGGCTCCAGGTTACCA | GCAACAACAAAAGGGAACAAA |
| MID785 | rs5873274 | 5q35.1 | TAGG | 81/85 | CTCTGGGCCTGTATGCAGAT | CCATTTCCCCTGCTACTCCT |
| MID1514 | rs2307710 | 6p12.3 | AGGA | 113/117 | TGCAGAGCCCATACCTACTG | GCACCTCTAAGTGGCTGAAGA |
| MID1484 | rs141366529 | 6p24.3 | CAAA | 84/88 | TCCCATGTATACCCACACCA | TTGAGTCTAACAGGAACAATTTCAA |
| MID1643 | rs2307839 | 6q22.1 | GA | 112/114 | ATCTGAGGTGGCATCATTCG | TCTTGCAAAATTAATCACACTCG |
| MID1945 | rs2308137 | 6q25.1 | GA | 93/95 | GAGTTTCCCGTTGAGCTTTG | AAAGGCCTGAAAGTCACGAA |
| MID1782 | rs2307978 | 7q21.11 | GA | 118/120 | TTCTCTCTGTGCATTTCTTTACG | GAACAAATTCCTAAGGGAACTAAGAA |
| MID1375 | rs72484380 | 7q33 | CTTTA | 91/96 | AAAGGGAAGCCATGTCTTTGT | AGAGTGACTGGGGTGGGAAC |
| MID3031 | rs5895447 | 8q24.23 | CA | 111/113 | GCCACTATCACTGGGGAGAT3 | GAAAGGACAGGGGGAACAGT |
| MID116 | rs16402 | 9p13.1 | TTAT | 91/95 | GCCTTTTTGGTTTTGGTGAA | CTGTATGGGGCTGGCTTTAG |
| MID1209 | rs2067294 | 9q21.11 | CTT | 80/83 | TATGCTATCCCGGCAATTCT | CGAGGAGTGAACAAGAAGCA |
| MID1384 | rs2307580 | 9q31.1 | AATT | 88/92 | GCAGCTTCCAACTGGTTCTT | TGGAACAGGACCATAGCATAA |
| MID520 | rs41293961 | 10p15.1 | CAA | 100/103 | TAGCATGGAAGGCTTTCAGA | CATCCCCCTCCTGAGTGAC |
| MID649 | rs79346574 | 10q21.1 | TAC | 79/82 | TTCCTTTTCCCATTGTGCTT | CCAGTCTACCCAAATGTATTCCA |
| MID3220 | rs34811743 | 11p14.1 | TG | 116/118 | ACACTTCGTACCCAGGATGC | GCCGTGAGAAAATGCTTCAT |
| MID3321 | rs33972805 | 11q24.2 | CT | 114 116 | CCAGAGCCATCTATCATTCTGA | CCACCAGCTCCATGCTTAGT |
| MID834 | rs1610919 | 12p12.3 | AT | 106/108 | TCATCACTTCCGTTTCCTTTC | GGTACTCGGCAAGAGAAATCA |
| MID1459 | rs67779220 | 12q24.33 | ATC | 114/117 | GCCTTGTCATCAAAATTCACC | CCGTCCACCTCTTCTGCTAC |
| MID1522 | rs2307718 | 13q31.13 | ACTG | 96/100 | AAAGGGACTATAATGAAAGCAAGA | AAGGGCAAAACAAAAACAGG |
| MID1997 | rs397696936 | 14q12 | AACTA | 107/112 | GGGAAGCAAGTTGGAAACAA | CTGCTCCTTTCACCTGGAAC |
| MID257 | rs16694 | 14q24.2 | GAG | 106/109 | TGAAAGAAACATGAGGGTGGT | TCTCCATGCTTTATGAATGCT |
| MID1824 | rs397720213 | 15q21.3 | TT | 68/70 | AAAACTAACAGGGTGCCACAA | CAAGTTGTGCTGTAAATGTAGCC |
| MID1323 | rs145913637 | 15q22.31 | TTCAAT | 80/85 | GAGACAGCACCTACCCCAAG | TTGGTGACAGGATAGAGAGCTG |
| MID2057 | rs2308249 | 16q21 | AC | 108/110 | TCTCCATTGGACAGACAGGA | GGAGGAGCAAGTGACCAAGA |
| MID1120 | rs2067208 | 16q24.1 | GCCAG | 111/116 | AGGTTGCTGGAGGCTTGAA | TCAGGCAGCTGAAGAGAATG |
| MID2648 | rs3051300 | 17p13.1 | GTAT | 94/98 | CCAAAGAATGTTGGGAATAACA | TTGCCCAGTCTCACACAATG |
| MID1436 | rs2307632 | 17q21.32 | GAA | 117/120 | CCTTAGGGCTGGGAAGATTG | TGTCCTAATTTGGCCATCTG |
| MID1823 | rs2308019 | 18p11.21 | CT | 87/89 | GGTGACAGGAGAGCTCCAAG | GAGCCAGAATGGCTGACTTT |
| MID3097 | rs34511541 | 18q12.2 | CTCTT | 115/120 | GGCCTTTATATGATGGGAAGGT | CCTTCTTAGGTTCAAAGACACTCA |
| MID2592 | rs36040336 | 19p13.3 | AT | 89/91 | GTTCTGCACGGGTTAACAGA | CTGCAACCTGTGAACCTCAA |
| MID1493 | rs2307689 | 19q13.31 | TTC | 97/100 | CCCCCTCAGATTAGCACCTT | CCCCTCGTTTGAAGAAGGA |
| MID1900 | rs361870 | 22q11.21 | TG | 72/74 | CTGAAGTGTGGTGGGCTTTC | ACAGGGACAGGCAGATGATG |
| MID2047 | rs397730557 | Xp22.31 | GA | 118/120 | AGCAGAAATTGACAGAACAAGTCA | TGCTAAAACGGAAAGAGCAC |
| ZFX/ZFY | / | X & Y | / | 87 | CAAGTGCTGGACTCAGATGTAACTG | TGAAGTAATGTCAGAAGCTAAAACATCA |
