# Supplementary material for: Measurement of fetal fraction in cell-free DNA from maternal plasma using a panel of insertion/deletion polymorphisms
Source: PLoS One. 2017 Oct 30;12(10):e0186771. doi: 10.1371/journal.pone.0186771 (PMC5662091; doi:10.1371/journal.pone.0186771)
Supplement: S2 Table — Twenty gDNA samples were sequenced and the number of homozygous and heterozygous markers was recorded. The ratio of allele A:B was calculated. Any indel with a median of greater than 0.01 for a homozygous indel or less than 0.9 for a heterozygous indel was excluded from further analysis. Additionally, indels yielding a median of fewer than 2,000 reads were also excluded. Excluded indels are shaded in grey. (DOCX) [file pone.0186771.s003.docx]

| **Indel** | **Number Homozygous** | **Median of Ratio Allele A:B** | **Number Heterozygous** | **Median of Ratio Allele A:B** |
| --- | --- | --- | --- | --- |
| MID2305 | 14 | 0.00 | 6 | 0.82 |
| MID187 | 10 | 0.00 | 10 | 0.96 |
| MID185 | 14 | 0.00 | 6 | 0.94 |
| MID668 | 11 | 0.00 | 9 | 0.89 |
| MID2050 | 14 | 0.00 | 6 | 0.95 |
| MID2045 | 12 | 0.00 | 8 | 0.93 |
| MID1372 | 9 | 0.00 | 11 | 0.93 |
| MID1830 | 16 | 0.00 | 4 | 0.91 |
| MID743 | 12 | 0.00 | 8 | 0.96 |
| MID785 | 12 | 0.00 | 8 | 0.93 |
| MID1514 | 12 | 0.01 | 8 | 0.94 |
| MID1643 | 12 | 0.01 | 8 | 0.97 |
| MID1945 | 5 | 0.01 | 15 | 0.95 |
| MID1782 | 10 | 0.00 | 10 | 0.94 |
| MID3031 | 12 | 0.00 | 8 | 0.94 |
| MID116 | 9 | 0.00 | 11 | 0.90 |
| MID1209 | 14 | 0.00 | 6 | 0.96 |
| MID1384 | 8 | 0.00 | 12 | 0.93 |
| MID520 | 13 | 0.00 | 7 | 0.95 |
| MID649 | 8 | 0.01 | 12 | 0.91 |
| MID3220 | 9 | 0.00 | 11 | 0.96 |
| MID3321 | 11 | 0.00 | 9 | 0.98 |
| MID834 | 6 | 0.08 | 14 | 0.94 |
| MID1522 | 11 | 0.00 | 9 | 0.97 |
| MID257 | 10 | 0.00 | 10 | 0.93 |
| MID1997 | 9 | 0.00 | 11 | 0.95 |
| MID1824 | 13 | 0.00 | 7 | 0.91 |
| MID1120 | 15 | 0.00 | 5 | 0.95 |
| MID2057 | 12 | 0.00 | 8 | 0.93 |
| MID2648 | 14 | 0.00 | 6 | 0.94 |
| MID1436 | 10 | 0.00 | 10 | 0.94 |
| MID3097 | 7 | 0.00 | 13 | 0.94 |
| MID1823 | 16 | 0.00 | 4 | 0.92 |
| MID2592 | 11 | 0.01 | 9 | 0.86 |
| MID1493 | 9 | 0.02 | 11 | 0.90 |
| MID1323 | 15 | 0.00 | 5 | 0.96 |
| MID1900 | 13 | 0.01 | 6 | 0.92 |
| MID2047 | 12 | 0.00 | 8 | 0.96 |
| MID1459 | 10 | 0.00 | 10 | 0.95 |
| MID1484 | 18 | 0.00 | 2 | 0.85 |
| MID770 | 11 | 0.00 | 9 | 0.91 |
| MID768 | 10 | 0.00 | 10 | 0.94 |
| MID1561 | 11 | 0.00 | 9 | 0.91 |
| MID1375 | 9 | 0.00 | 11 | 0.95 |
| ZFX_ZFY | 20 | 0.00 | 0 | 0 |
