# Supplementary material for: Measurement of fetal fraction in cell-free DNA from maternal plasma using a panel of insertion/deletion polymorphisms
Source: PLoS One. 2017 Oct 30;12(10):e0186771. doi: 10.1371/journal.pone.0186771 (PMC5662091; doi:10.1371/journal.pone.0186771)
Supplement: S3 Table — Figures in brackets show the number of informative indels for each sample. (DOCX) [file pone.0186771.s004.docx]

| **Sample** | **Gestation (weeks)** | **PCR-free %** | **ThruPLEX %** | **Nano %** |
| --- | --- | --- | --- | --- |
| 1 | 12+6 | 17.9 (15) | 20.2 (15) | 19.3 (15) |
| 2 | 23+0 | 11.7.0 (14) | 13.9 (16) | 15.7 (16) |
| 3 | 12+1 | 30.3 (12) | 33.2 (13) | 30.1 (12) |
| 4 | 24+0 | 15.5 (13) | 19.4 (13) | 18.9 (14) |
| 5 | 21+6 | 17.6 (13) | 17.3 (14) | 17.2 (14) |
| 6 | 15+0 | 30.6 (11) | 32.2 (12) | 31.8 (12) |
