# Supplementary material for: Measurement of fetal fraction in cell-free DNA from maternal plasma using a panel of insertion/deletion polymorphisms
Source: PLoS One. 2017 Oct 30;12(10):e0186771. doi: 10.1371/journal.pone.0186771 (PMC5662091; doi:10.1371/journal.pone.0186771)
Supplement: S4 Table — (DOCX) [file pone.0186771.s005.docx]

| **Sample No.** | **WGS (%)** | **Indels (%)** | **ZFX/ZFY (%)** |
| --- | --- | --- | --- |
| 2 | 23.2 | 28.3 | 31.6 |
| 5 | 5.8 | 5.2 | 2.3 |
| 6 | 4.9 | 4.7 | 3.3 |
| 7 | 4.6 | 2.9 | 3.7 |
| 14 | 6.8 | 5.1 | 2.9 |
| 15 | 6.1 | 8.7 | 6.2 |
| 21 | 5.8 | 4.9 | 1.7 |
| 22 | 9.5 | 5.5 | 6.4 |
| 23 | 11.3 | 8.8 | 9.1 |
| 24 | 2.9 | 8.6 | 4.3 |
| 30 | 10.4 | 10.4 | 0.5 |
| 31 | 11.5 | 11.0 | 6.6 |
| 32 | 11.1 | 17.2 | 2.1 |
| 40 | 15.5 | 19.9 | 33.6 |
| 42 | 15.6 | 10.2 | 14.9 |
| 43 | 16.9 | 19.1 | 12.8 |
| 44 | 10.4 | 8.9 | 9.2 |
| 45 | 5.7 | 4.6 | 3.3 |
| 46 | 5.1 | 5.8 | 4.3 |
| 47 | 10.4 | 9.0 | 8.8 |
| 53 | 9.4 | 5.3 | 4.6 |
| 54 | 10.8 | 7.4 | 5.5 |
| 55 | 14.4 | 8.0 | 11.3 |
| 56 | 9.3 | 8.5 | 6.3 |
| 57 | 5.5 | 6.9 | 3.4 |
| 58 | 6.9 | 5.7 | 3.7 |
| 59 | 11.7 | 7.5 | 7.6 |
| 60 | 0.5 | 2.2 | 1.4 |
| 71 | 10.4 | 11.0 | 8.1 |
| 72 | 12.6 | 8.2 | 13.4 |
| 73 | 8.2 | 10.0 | 1.0 |
| 74 | 10.0 | 4.7 | 6.7 |
| 75 | 7.7 | 4.7 | 5.1 |
| 76 | 5.9 | 6.9 | 8.2 |
| 77 | 10 | 8.5 | 8.7 |
| 78 | 12.7 | 8.4 | 7.8 |
| 80 | 7.4 | 12.9 | 13.1 |
| 84 | 8.0 | 9.9 | 7.1 |
| 85 | 5.0 | 6.9 | 9.5 |
| 86 | 16.9 | 25.4 | 5.6 |
| 87 | 9.7 | 8.8 | 8.2 |
| 88 | 12.8 | 26.4 | 2.9 |
| 93 | 28.2 | 21.3 | 15.5 |
| 95 | 22.7 | 17.2 | 7.1 |
| 97 | 8.0 | 6.9 | 5.6 |
| 98 | 14.4 | 9.2 | 6.7 |
| 99 | 7.3 | 11.1 | 6.0 |
| 100 | 12.6 | 12.3 | 4.8 |
| 101 | 8.3 | 23.7 | 7.0 |
| 112 | 11.0 | 17.8 | 4.2 |
| 113 | 3.1 | 6.0 | 3.5 |
| 114 | 9.5 | 8.3 | 6.6 |
| 120 | 2.6 | 10.3 | 5.3 |
| 127 | 18.4 | 17.6 | 4.1 |
| 130 | 34.8 | 22.8 | 31.5 |
| 137 | 12.6 | 10.8 | 13.3 |
| 138 | 12.6 | 10.9 | 3.5 |
| 139 | 22.2 | 11.4 | 12.5 |
| 149 | 21.7 | 13.1 | 6.9 |
| 150 | 9.8 | 16.8 | 6.6 |
| 155 | 12.5 | 27.8 | 16.4 |
