# Supplementary material for: Measurement of fetal fraction in cell-free DNA from maternal plasma using a panel of insertion/deletion polymorphisms
Source: PLoS One. 2017 Oct 30;12(10):e0186771. doi: 10.1371/journal.pone.0186771 (PMC5662091; doi:10.1371/journal.pone.0186771)
Supplement: S5 Table — ‘-‘ indicates that the data was not collected. Samples highlighted in grey were also used for the comparison of methods used for measuring fetal fraction. Samples with ‘*’ were used to compare the library prep kits. (DOCX) [file pone.0186771.s006.docx]

| **Sample number** | **Gestation (weeks)** | **Ethnicity** | **Fetal Sex** | **No. of Informative Indels** | **Fetal Fraction (%)** |
| --- | --- | --- | --- | --- | --- |
| 1 | 25+2 | Chinese | Female | 3 | 13.5 |
| 2 | 18+2 | Chinese | Male | 3 | 28.3 |
| 3 | 23+4 | Chinese | Male | 3 | 38.4 |
| 4 | 18+5 | Chinese | Female | 4 | 6.1 |
| 5 | 16+5 | Other | Male | 4 | 5.2 |
| 6 | - | Other | Male | 4 | 4.7 |
| 7 | - | Other | Male | 4 | 2.9 |
| 8 | 18+3 | Chinese | Female | 5 | 3.5 |
| 9 | - | Chinese | Female | 5 | 11.2 |
| 10 | 21+1 | Chinese | Female | 5 | 21.9 |
| 11 | 17+6 | Chinese | Male | 5 | 15.0 |
| 12 | 12+4 | Malay | Male | 6 | 4.2 |
| 13 | 12+4 | Indian | Male | 6 | 9.3 |
| 14 | - | Other | Male | 6 | 5.1 |
| 15 | 13+1 | Chinese | Male | 6 | 8.7 |
| 16 | 29+0 | Chinese | Female | 7 | 27.6 |
| 17 | 27+1 | Indian | Female | 7 | 24.4 |
| 18 | 20+0 | Chinese | Female | 7 | 11.7 |
| 19 | 26+0 | Indian | Male | 7 | 26.1 |
| 20 | 21+5 | Indian | Female | 7 | 21.0 |
| 21 | 15+6 | Indian | Male | 7 | 4.9 |
| 22 | 10+4 | Chinese | Male | 7 | 5.5 |
| 23 | 12+1 | Other | Male | 7 | 8.8 |
| 24 | 12+0 | Other | Male | 7 | 8.6 |
| 25 | 21+0 | Indian | Female | 8 | 6.2 |
| 26 | 29+4 | Chinese | Female | 8 | 12.8 |
| 27 | - | Chinese | Female | 8 | 7.9 |
| 28 | - | Chinese | Female | 8 | 9.6 |
| 29 | 33+0 | Indian | Female | 8 | 23.4 |
| 30 | 13+5 | Indian | Male | 8 | 10.4 |
| 31 | 12+3 | Indian | Male | 8 | 11.0 |
| 32 | 12+5 | Chinese | Male | 8 | 17.2 |
| 33 | 24+0 | Other | Female | 9 | 19.4 |
| 34 | 24+3 | Chinese | Male | 9 | 6.9 |
| 35 | 22+0 | Chinese | Female | 9 | 17.4 |
| 36 | 21+5 | Chinese | Female | 9 | 9.5 |
| 37 | 22+4 | Chinese | Male | 9 | 5.9 |
| 38 | 18+3 | Chinese | Male | 9 | 4.2 |
| 39 | - | Chinese | Male | 9 | 10.1 |
| 40 | 24 | Indian | Male | 9 | 19.9 |
| 41 | 15+4 | Chinese | Male | 9 | 19.8 |
| 42 | 22+0 | Chinese | Male | 9 | 10.2 |
| 43 | 11+5 | Chinese | Male | 9 | 19.1 |
| 44 | - | Other | Male | 9 | 8.9 |
| 45 | - | Other | Male | 9 | 4.6 |
| 46 | - | Other | Male | 9 | 5.8 |
| 47 | 11+0 | Malay | Male | 9 | 9.0 |
| 48 | 21+5 | Other | Male | 10 | 28.1 |
| 49 | 10+4 | Chinese | Female | 10 | 9.6 |
| 50 | 20+2 | Chinese | Female | 10 | 12.4 |
| 51 | 12+0 | Other | Female | 10 | 24.2 |
| 52 | 12+3 | Indian | Female | 10 | 23.0 |
| 53 | 18+0 | Other | Male | 10 | 5.3 |
| 54 | 12+1 | Chinese | Male | 10 | 7.4 |
| 55 | 13+1 | Chinese | Male | 10 | 8.0 |
| 56 | 20+4 | Chinese | Male | 10 | 8.5 |
| 57 | 12+2 | Chinese | Male | 10 | 6.9 |
| 58 | 12+1 | Chinese | Male | 10 | 5.7 |
| 59 | 12+5 | Other | Male | 10 | 7.5 |
| 60 | 11+4 | Indian | Male | 10 | 2.2 |
| 61 | 12+0 | Chinese | Male | 11 | 22.8 |
| 62 | 26+1 | Malay | Male | 11 | 14.7 |
| 63 | 23+1 | Chinese | Male | 11 | 8.6 |
| 64 | 22+2 | Chinese | Male | 11 | 5.6 |
| 65 | 10+0 | Other | Male | 11 | 22.5 |
| 66 | 16+3 | Indian | Female | 11 | 19.7 |
| 67 | 23+5 | Chinese | Male | 11 | 25.0 |
| 68 | 12+1 | Chinese | Male | 11 | 19.5 |
| 69 | 22+4 | Chinese | Male | 11 | 6.1 |
| 70 | 20+2 | Chinese | Male | 11 | 17.6 |
| 71 | 12+0 | Chinese | Male | 11 | 11.0 |
| 72 | 12+6 | Other | Male | 11 | 8.2 |
| 73 | 16+1 | Chinese | Male | 11 | 10.0 |
| 74 | 29+0 | Indian | Male | 11 | 4.7 |
| 75 | 13+5 | Chinese | Male | 11 | 4.7 |
| 76 | 12+1 | Other | Male | 11 | 6.9 |
| 77 | 13+0 | Chinese | Male | 11 | 8.5 |
| 78 | 10+6 | Chinese | Male | 11 | 8.4 |
| 79 | 12+6 | Chinese | Male | 12 | 13.8 |
| 80 | 12+1 | Chinese | Male | 12 | 12.9 |
| 81* | 12+1 | Other | Male | 12 | 30.1 |
| 82* | 15+0 | Chinese | Female | 12 | 31.8 |
| 83 | 12+2 | Indian | Female | 12 | 11.0 |
| 84 | 19+4 | Chinese | Male | 12 | 9.9 |
| 85 | 16+1 | Chinese | Male | 12 | 6.9 |
| 86 | 12+4 | Chinese | Male | 12 | 25.4 |
| 87 | 11+3 | Indian | Male | 12 | 8.8 |
| 88 | 24+5 | Chinese | Male | 13 | 26.4 |
| 89 | 12+0 | Malay | Female | 13 | 15.1 |
| 90 | 18+4 | Indian | Female | 13 | 20.2 |
| 91 | 20+0 | Chinese | Male | 13 | 6.3 |
| 92 | - | Chinese | Male | 13 | 12.7 |
| 93 | 35+4 | Malay | Male | 13 | 21.3 |
| 94 | 39+3 | Other | Male | 13 | 17.5 |
| 95 | 27+0 | Indian | Male | 13 | 17.2 |
| 96 | 12+2 | Chinese | Male | 13 | 16.8 |
| 97 | 15+5 | Indian | Male | 13 | 6.9 |
| 98 | 12+3 | Other | Male | 13 | 19.1 |
| 99 | 12+6 | Chinese | Male | 13 | 11.1 |
| 100 | 13+0 | Chinese | Male | 13 | 12.3 |
| 101 | 12+3 | indian | Male | 14 | 23.7 |
| 102 | 13+0 | Chinese | Male | 14 | 17.7 |
| 103 | 21+1 | Chinese | Female | 14 | 8.4 |
| 104 | - | Chinese | Female | 14 | 17.6 |
| 105 | 34+3 | Malay | Female | 14 | 10.5 |
| 106 | 35+0 | Malay | Female | 14 | 17.9 |
| 107* | 24+0 | Indian | Female | 14 | 18.9 |
| 108* | 21+6 | Chinese | Male | 14 | 17.2 |
| 109 | 28+0 | Other | Male | 14 | 12.4 |
| 110 | 12+6 | Other | Male | 14 | 17.8 |
| 111 | 11+6 | Other | Male | 14 | 15.3 |
| 112 | - | Chinese | Male | 14 | 17.8 |
| 113 | - | Other | Male | 14 | 6.0 |
| 114 | 13+6 | Other | Male | 14 | 8.3 |
| 115 | 20+4 | Malay | Female | 15 | 20.8 |
| 116 | 35+0 | Indian | Male | 15 | 28.0 |
| 117 | 26+5 | Indian | Female | 15 | 24.0 |
| 118 | 12+3 | Other | Female | 15 | 16.3 |
| 119 | - | Chinese | Female | 15 | 5.7 |
| 120 | 8+0 | Malay | Male | 15 | 10.3 |
| 121 | 31+5 | Chinese | Female | 15 | 14.9 |
| 122 | 12+4 | Other | Male | 15 | 22.1 |
| 123 | 11+6 | Other | Female | 15 | 27.3 |
| 124 | 12+4 | Chinese | Female | 15 | 19.6 |
| 125 | 12+3 | Chinese | Female | 15 | 22.2 |
| 126 | 14+0 | Chinese | Male | 15 | 15.7 |
| 127 | 19+0 | Other | Male | 15 | 17.6 |
| 128* | 12+6 | Indian | Female | 15 | 19.3 |
| 129 | 37+0 | Other | Male | 16 | 17.9 |
| 130 | 38+2 | Other | Male | 16 | 22.8 |
| 131 | 25+0 | Chinese | Male | 16 | 16.4 |
| 132 | 39+4 | Indian | Female | 16 | 10.1 |
| 133 | 26 | Malay | Female | 16 | 22.4 |
| 134* | 23+0 | Malay | Male | 16 | 15.7 |
| 135 | 26+3 | Indian | Male | 16 | 28.9 |
| 136 | 21+3 | Indian | Female | 16 | 16.2 |
| 137 | 12+3 | Other | Male | 16 | 10.8 |
| 138 | 21+3 | Other | Male | 16 | 10.9 |
| 139 | 28+5 | Indian | Male | 16 | 11.4 |
| 140 | 12+4 | Indian | Female | 17 | 21.4 |
| 141 | 10+2 | Indian | Female | 17 | 20.8 |
| 142 | - | Chinese | Female | 17 | 9.8 |
| 143 | 24+2 | Chinese | Female | 17 | 14.2 |
| 144 | 11+0 | Chinese | Female | 17 | 10.4 |
| 145 | 20+0 | Chinese | Female | 18 | 14.6 |
| 146 | 32+0 | Malay | Female | 18 | 10.2 |
| 147 | 19+0 | Indian | Female | 18 | 17.0 |
| 148 | 28+0 | Other | Female | 18 | 22.5 |
| 149 | 34+0 | Chinese | Male | 18 | 13.1 |
| 150 | 13+5 | Other | Male | 18 | 16.8 |
| 151 | 32+0 | Malay | Male | 19 | 24.0 |
| 152 | 21+4 | Chinese | Male | 19 | 8.1 |
| 153 | 28+6 | Chinese | Female | 19 | 16.4 |
| 154 | 19+2 | Chinese | Male | 19 | 7.4 |
| 155 | 23+0 | Indian | Male | 19 | 27.8 |
| 156 | 12+1 | Chinese | Male | 20 | 17.7 |
| 157 | 13+1 | Indian | Female | 21 | 16.6 |
