# Supplementary material for: Measurement of fetal fraction in cell-free DNA from maternal plasma using a panel of insertion/deletion polymorphisms
Source: PLoS One. 2017 Oct 30;12(10):e0186771. doi: 10.1371/journal.pone.0186771 (PMC5662091; doi:10.1371/journal.pone.0186771)
Supplement: S7 Table — Total sample number, n = 157. (DOCX) [file pone.0186771.s008.docx]

| **Indel** | **Informative in n cases** | **Percentage Informativity (%)** |
| --- | --- | --- |
| MID187 | 42 | 26.8 |
| MID2050 | 49 | 31.2 |
| MID2045 | 48 | 30.6 |
| MID1372 | 53 | 33.8 |
| MID1830 | 74 | 47.1 |
| MID785 | 69 | 43.9 |
| MID1514 | 65 | 41.4 |
| MID1643 | 60 | 38.2 |
| MID1945 | 71 | 45.2 |
| MID1782 | 50 | 31.8 |
| MID3031 | 57 | 36.3 |
| MID116 | 42 | 26.8 |
| MID1209 | 49 | 31.2 |
| MID1384 | 42 | 26.8 |
| MID520 | 59 | 37.6 |
| MID649 | 40 | 25.5 |
| MID3220 | 59 | 37.6 |
| MID3321 | 70 | 44.6 |
| MID1522 | 62 | 39.5 |
| MID257 | 44 | 28.0 |
| MID1997 | 47 | 29.9 |
| MID1824 | 72 | 45.9 |
| MID1120 | 64 | 40.8 |
| MID2057 | 45 | 28.7 |
| MID2648 | 39 | 24.8 |
| MID1436 | 35 | 22.3 |
| MID3097 | 40 | 25.5 |
| MID1323 | 24 | 15.3 |
| MID1900 | 68 | 43.3 |
| MID2047 | 39 | 24.8 |
| MID1459 | 40 | 25.5 |
| MID770 | 45 | 28.7 |
| MID768 | 69 | 43.9 |
| MID1561 | 64 | 40.8 |
| MID1375 | 46 | 29.3 |
