# Supplementary figures and images for: Measurement of fetal fraction in cell-free DNA from maternal plasma using a panel of insertion/deletion polymorphisms
Source: PLoS One. 2017 Oct 30;12(10):e0186771. doi: 10.1371/journal.pone.0186771 (PMC5662091; doi:10.1371/journal.pone.0186771)

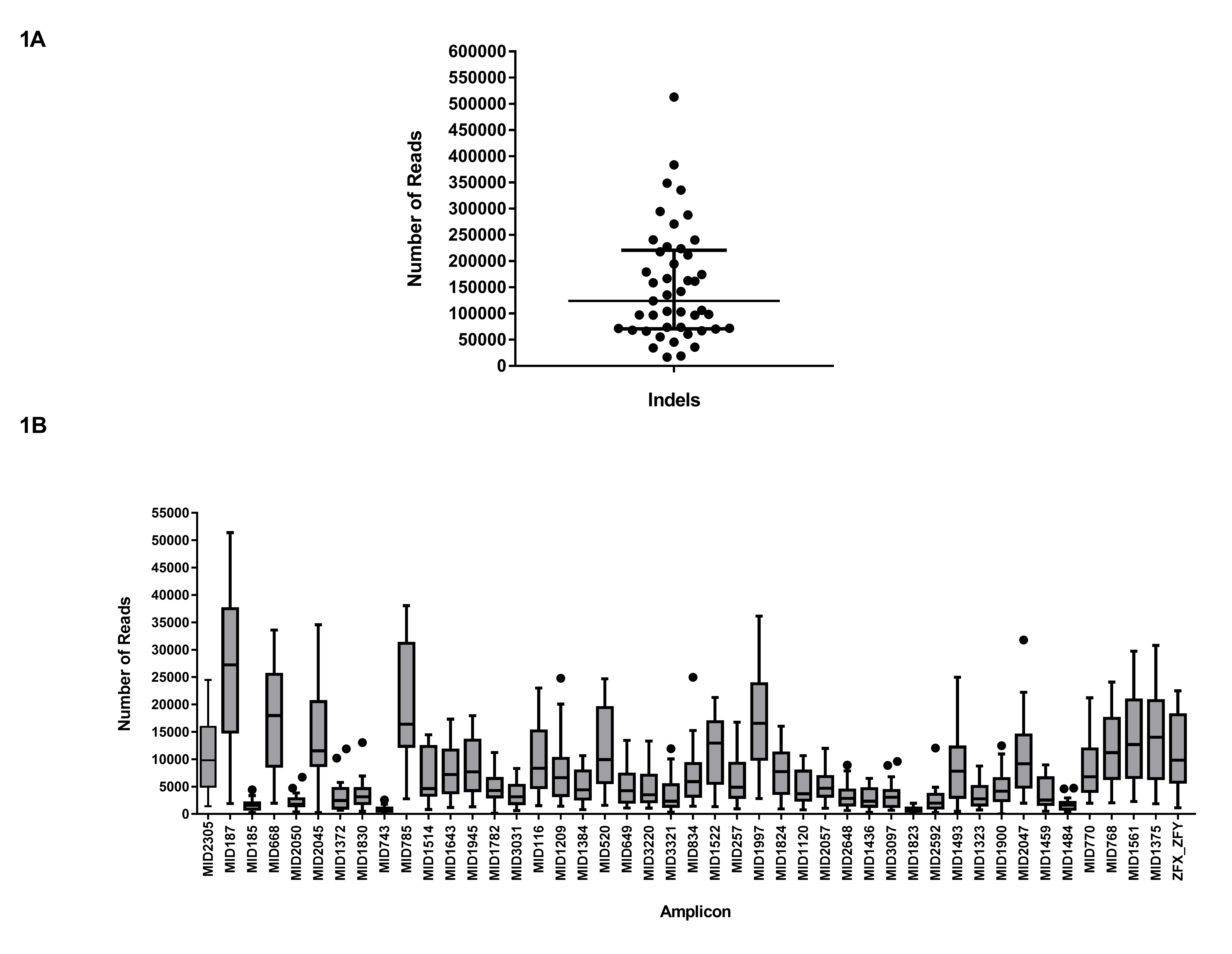

Supplement: S1 Fig — (A) Number of reads generated using the indel panel for each gDNA sample. The median and interquartile range are indicated; (B) A Tukey box plot showing median number of reads per indel amplicon. Median and interquartile range are shown by grey boxes, median is shown by the horizontal line, and whiskers represent the range of the data. Outliers are indicated by black circles. (TIF) [file pone.0186771.s009.tif]

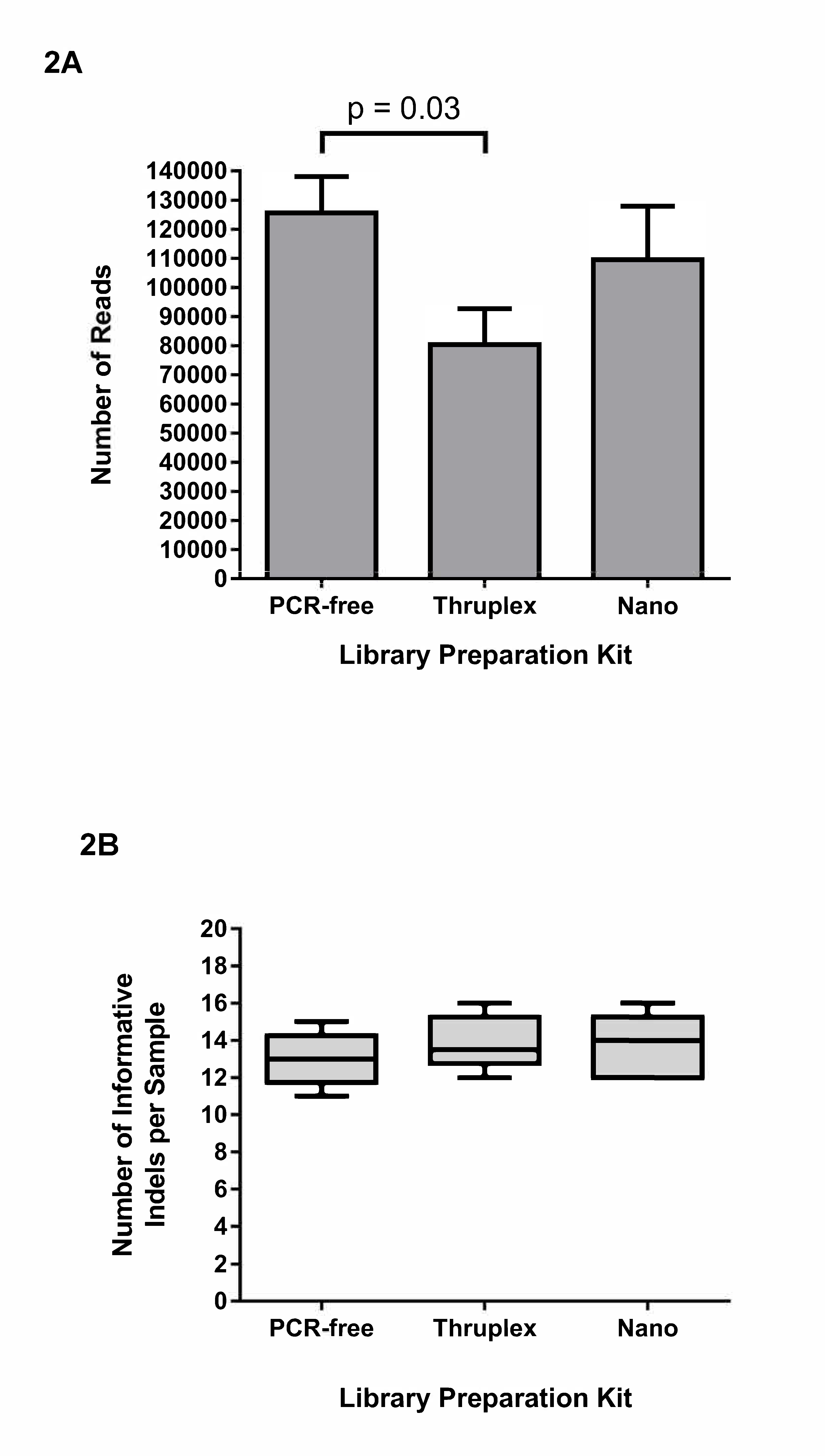

Supplement: S2 Fig — Three kits were compared, namely the PCR-free DNA Sample Preparation kit, the TruSeq Nano DNA Sample Preparation kit, and the ThruPLEX kit. A) There is a significantly higher number of reads from the PCR-free kit compared to Thruplex; B) No difference was seen between the kits in number of informative indels; C) No difference was seen in the fetal fraction for any of the six samples between the three kits. Each dot represents an individual indel. Horizontal bars represent the mean estimation of fetal fraction, and the standard error of the mean (SEM) is shown for each sample. (TIF) [file pone.0186771.s010.tif]

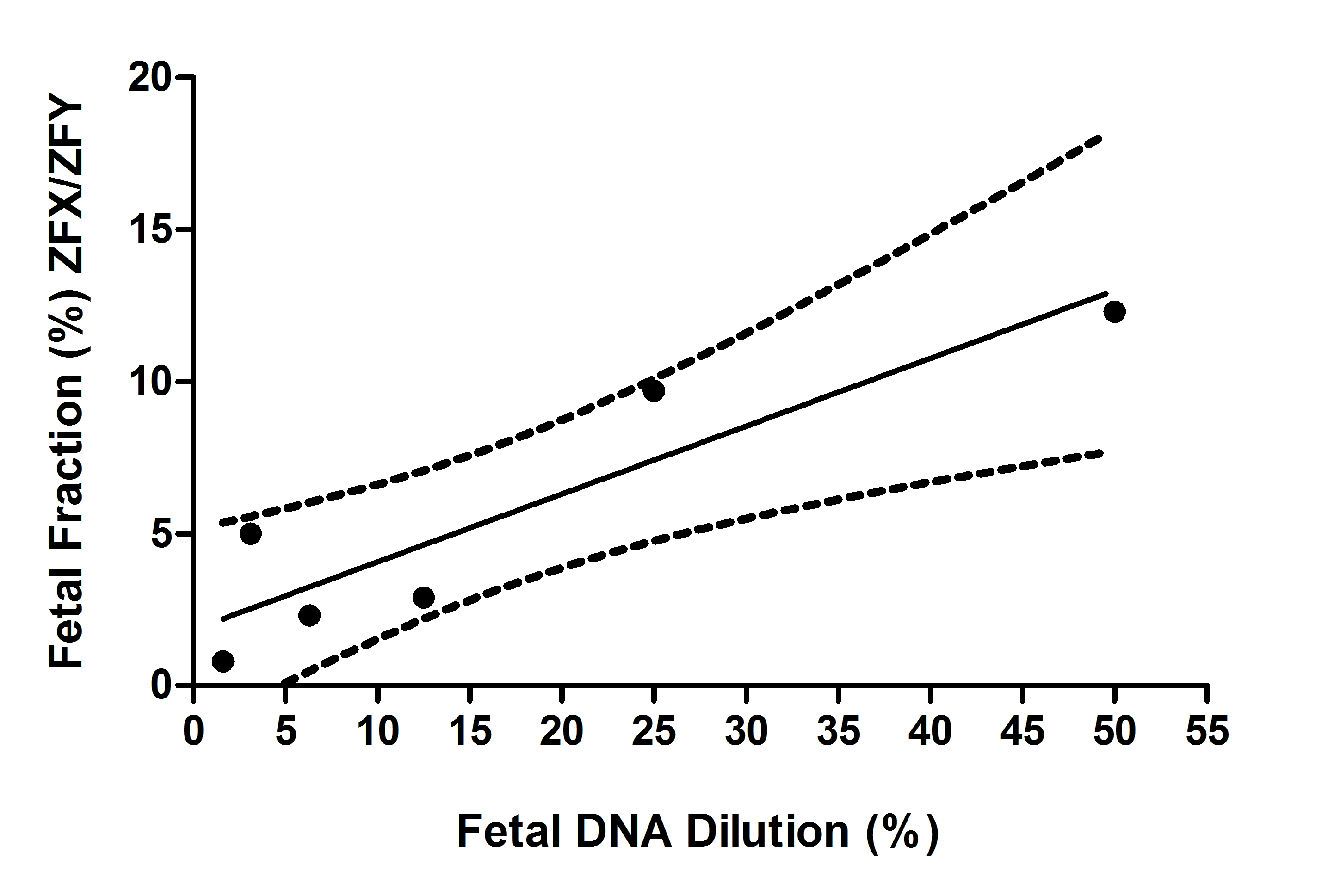

Supplement: S3 Fig — Slope = 0.22 (0.08–0.36), r2 = 0.83, p = 0.012. The 95% confidence intervals of the slope are represented by dotted lines. (TIF) [file pone.0186771.s011.tif]

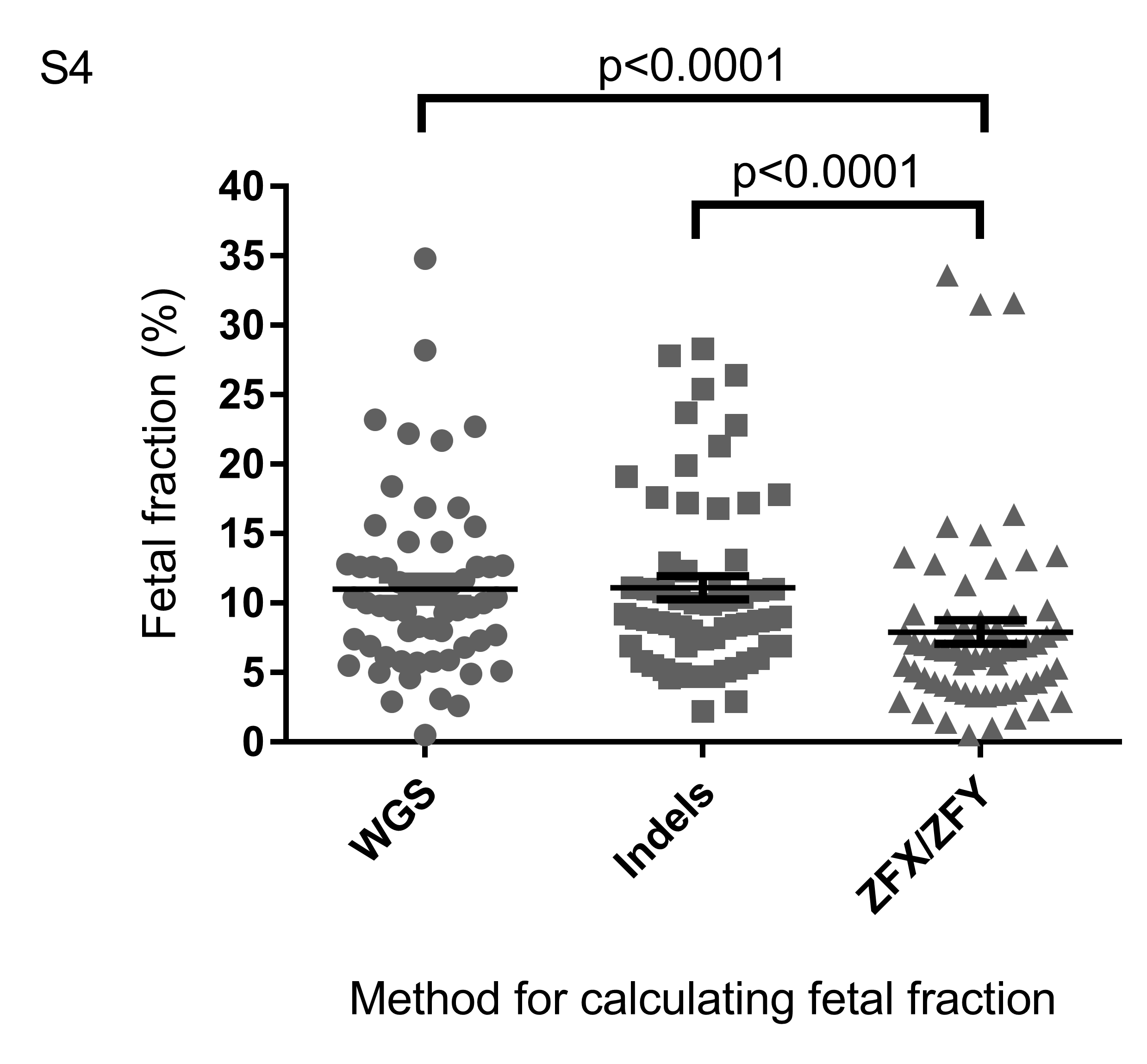

Supplement: S4 Fig — (TIF) [file pone.0186771.s012.tif]

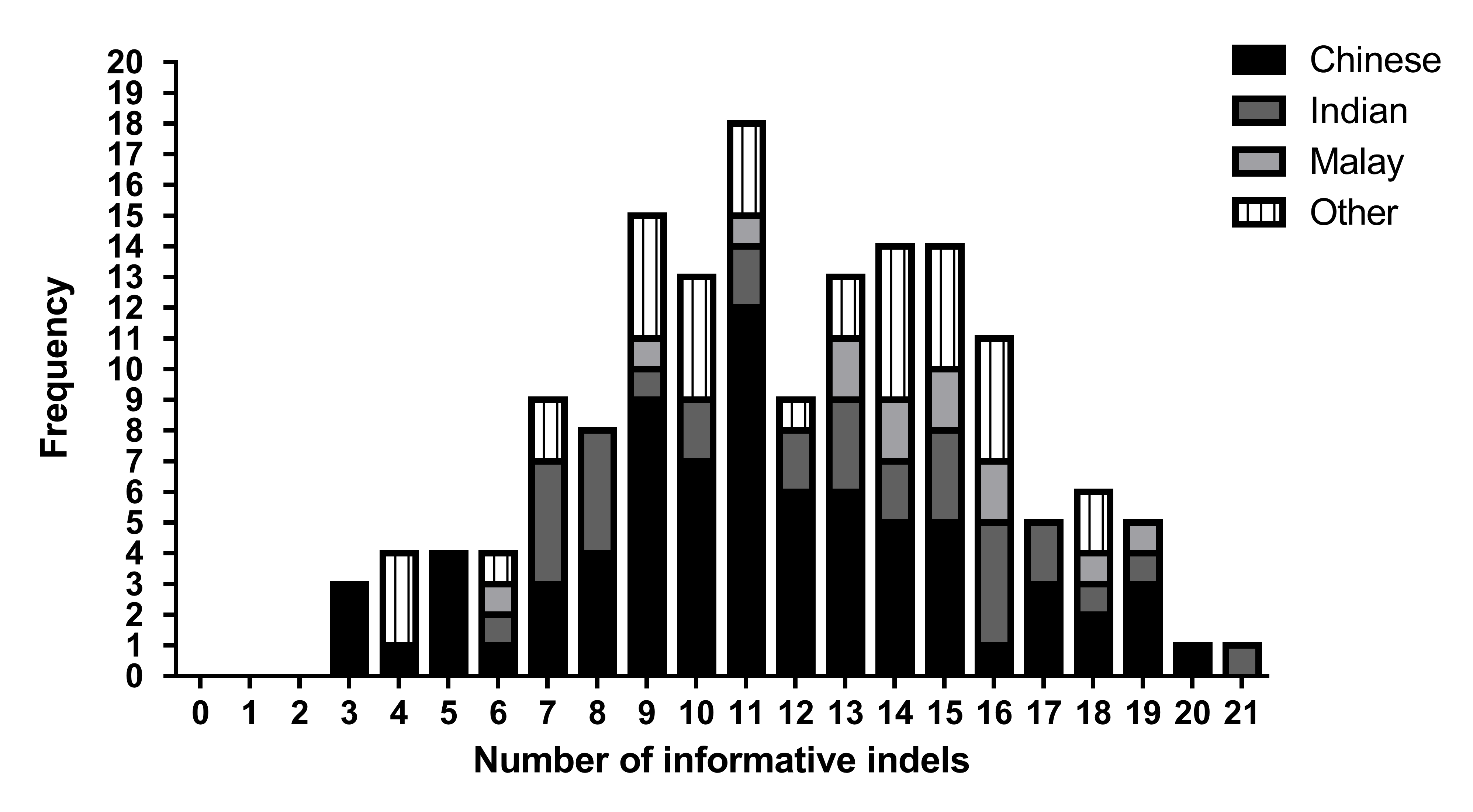

Supplement: S5 Fig — Ethnicity for all 157 patients was recorded: Chinese, n = 76; Malay, n = 13; Indian, n = 33; Others, n = 35. (TIF) [file pone.0186771.s013.tif]
